# Development of moringa seed powder-modified slag geopolymers for enhanced mechanical properties and effective dye removal

**DOI:** 10.1038/s41598-025-91091-z

**Published:** 2025-03-15

**Authors:** Soher A. Hashish, Essam A. Kishar, Doaa A. Ahmed, Sheren M. Ragei, Aya Allah M. Ebrahim

**Affiliations:** Chemistry Department, Faculty of Women for Arts, Science and Education, Ain Shams University, Cairo, 11757 Egypt

**Keywords:** Geopolymer, Moringa seeds, Slag, Crystal Violet, Adsorption comparative study, Environmental chemistry, Environmental impact, Engineering, Materials science

## Abstract

Crystal violet (CV), a widely used dye in paints and textiles, poses a significant environmental threat due to its non-biodegradable nature. A modified slag-based geopolymer has been developed to address this issue by incorporating raw moringa seed powder (MSP), an agricultural waste. The geopolymers (SM1, SM2, and SM3) were created by adding different percentages of MSP (0.2%, 0.6%, and 1% by weight) to ground granulated blast furnace slag (GGBFS), using sodium silicate and 10 M sodium hydroxide as alkali activators. This combination enhances the geopolymer’s mechanical and adsorbent properties, making it more effective for CV removal. The geopolymer composites were analyzed using X-ray diffraction (XRD), Fourier-transform infrared spectroscopy (FTIR), and scanning electron microscopy (SEM). Their mechanical properties were evaluated by conducting compressive strength and total porosity tests. Pore structure analysis was performed using nitrogen adsorption and desorption techniques, and the point of zero charges was determined. Additionally, batch experiments were carried out to investigate the adsorption of CV dye, employing two isotherm models and kinetic models for analysis. The SM1 mix, which is a modified slag-based geopolymer containing 0.2% MSP, exhibited the highest compressive strength at 73 MPa after 180 days, representing a 25.8% improvement compared to the control mix (100% slag). Furthermore, modified geopolymer mixes showed greater adsorption activity toward crystal violet compared to the control mix, with the SM3 mix achieving an adsorption capacity of up to 322.58 mg/g. The study demonstrates that adding MSP to slag-based geopolymer enhances mechanical strength and adsorption capacity. This indicates a positive impact on the composite’s surface properties and highlights the environmental benefits of utilizing industrial and agricultural waste in wastewater treatment.

## Introduction

Geopolymer is an inorganic polymer obtained by polymerizing aluminosilicate material, which can be of geological origin (e.g. kaolin, bentonites, rice husk ash) or industrial waste (e.g., slag, ash or red mud, or recycled glass) using alkaline friendly agents (e.g. NaOH or KOH, soluble silicates). The geopolymerization process involves different stages: Releasing of silicate and aluminate, gelation, polymerization, and hardening^1^. Blast furnace slag is a highly reactive aluminosilicate material formed in the processes of iron manufacture from iron ore. It has been used extensively in the production of geopolymers^2^. Recently there has been a lot of interest in the addition of organic polymer to geopolymer because of their ability to fill the gaps in the pores, bridge the small cracks, and create a cross-intersecting network with the hydrates of cement, hence increasing the strength^3–5^. The addition of organic polymer (up to 5) to the slag geopolymer matrix provides more binding locations that generate a three-dimensional structure when reacting with C-A-S-H. Moreover, they decrease porosity and increase compressive strength^5^. Moringa Oleifera powder has been used to enhance natural polymers that are ‘greener’ than other chemical polymers to prepare Polymer-modified concrete to increase the durability and sustainability of concrete. When Moringa Oleifera is used in place of cement in natural polymer-modified mortar, it improves the mortar’s strength, bonding mechanism, and durability in brackish and marine environments. 0.2% of the weight of cement^6^. According to Elinwa, the examination of the experimental data gathered on Moringa olifera seed powder (MOSP) and MOSP-concrete demonstrated that MOSP is considerably silicate (Quartz and Cristobalite), and that using MOSP in place of cement results in high-quality concrete with an ideal replacement percentage of 0.2% wt% of cement^7^. Moringa Oleifera (MO) Plants commonly found in tropical and subtropical climates. Several studies have been conducted to understand the chemical composition of Moringa Oleifera seeds^8,9^. They have demonstrated that moringa seeds contain 84% oleic acid^10^. Because of the variety of function groups (such as phosphate, carboxyl, and amino acids), MO was used as a natural coagulating agent^11^. It is thought that moringa oleifera seeds can provide ecologically, friendly biosorbent with a reasonable cost for water^12^. Water pollution is largely affected by dyes, whose presence causes aesthetic pollution. The dyes’ decreased biodegradability causes them to hinder photosynthesis by reducing light penetration^13^. Consequently, to cure the water and eventually utilize these unconventional fluids, it will be necessary to remove these organic compounds from the aquatic ecosystem^14^. A water-soluble cationic colorant called crystal violet, often known as gentian violet, imparts a violet hue to aqueous solutions^15^. It is widely used in the textile industry, producing paints and printing^16^. unluckily, crystal violet has cancer-causing material and is regarded as a challenging molecule to control because of its resistivity to microbial deterioration and capability to survive in a wide range of environmental conditions^17^.

Dyes can be removed from water by filtration^18^, oxidation^19^ coagulation-flocculation^20^, precipitation chemical^21^, ion exchange^22^, catalysis^23^ and adsorption. Adsorption’s accessibility, affordability, and sensitivity to toxic compounds make it an effective method for removing dyes from water^24^, and the possibility of regenerating used sorbents^25^. Crystal violet removal has been studied by various researchers using different adsorbents such as clays^26,27^ molecular sieves^28^, magnetic adsorbents^29^, biosorbent materials^30^, carbon^31^, silica^32^, raw chitin and multifunctional membrane^14^. Recently, there has been increasing concerns about the utilization of eco-friendly materials as adsorbents such as geopolymer. Geopolymers have a porous structure with cation exchange characteristics, enabling them to remove pollutants (metals and dyes) from water media^31,33^. A mesoporous geopolymer was prepared using metakaolin and rice husk ash to remove crystal violet dye from aqueous solutions^34^. After 120 min, the adsorption equilibrium was reached, and 276.9 mg g^–1^ was the maximum adsorption capacity. Purbasar, A. et al.^35^ used fly ash-based geopolymer and the maximum adsorption capacity of CV dyes was 45.45 mg g^− 1^. The introduction of Moringa seeds powder (MSP) in geopolymer research is noteworthy due to its distinct properties and the fresh perspective it offers. Contrary to traditional techniques, this study is the first to examine the possibility of MSP as an additional material in geopolymer systems. Unlike conventional pozzolanic materials or inert fillers, the organic components of MSP may interact chemically with the geopolymer matrix, potentially leading to new reaction pathways and improved mechanical and adsorption properties.

The primary focus of this research is to develop modified geopolymer pastes using industrial waste (slag) with the incorporation of moringa seed powder to enhance their mechanical properties. Furthermore, the study aims to explore the adsorption behavior of crystal violet dye by the modified green geopolymer obtained from industrial and agricultural wastes. This innovative approach not only aims to improve the mechanical properties of geopolymer pastes but also seeks to provide a sustainable solution for waste management in construction materials. In this investigation, we formulated geopolymer cement pastes from slag with varying proportions of moringa seed powder (0, 0.2, 0.6, and 1 wt%). We used a 2.5:1 ratio of sodium silicate to sodium hydroxide as the alkali activator. The hydration properties of the green geopolymer cement pastes were assessed after being cured for various durations ranging from 3 to 180 days at room temperature under 100% humidity. We also looked into the effects of various factors on the adsorption behavior of differently prepared geopolymer mixes, kinetics, and the type of adsorption model.

## Materials and methodology

### Materials

**Fig. 1:**
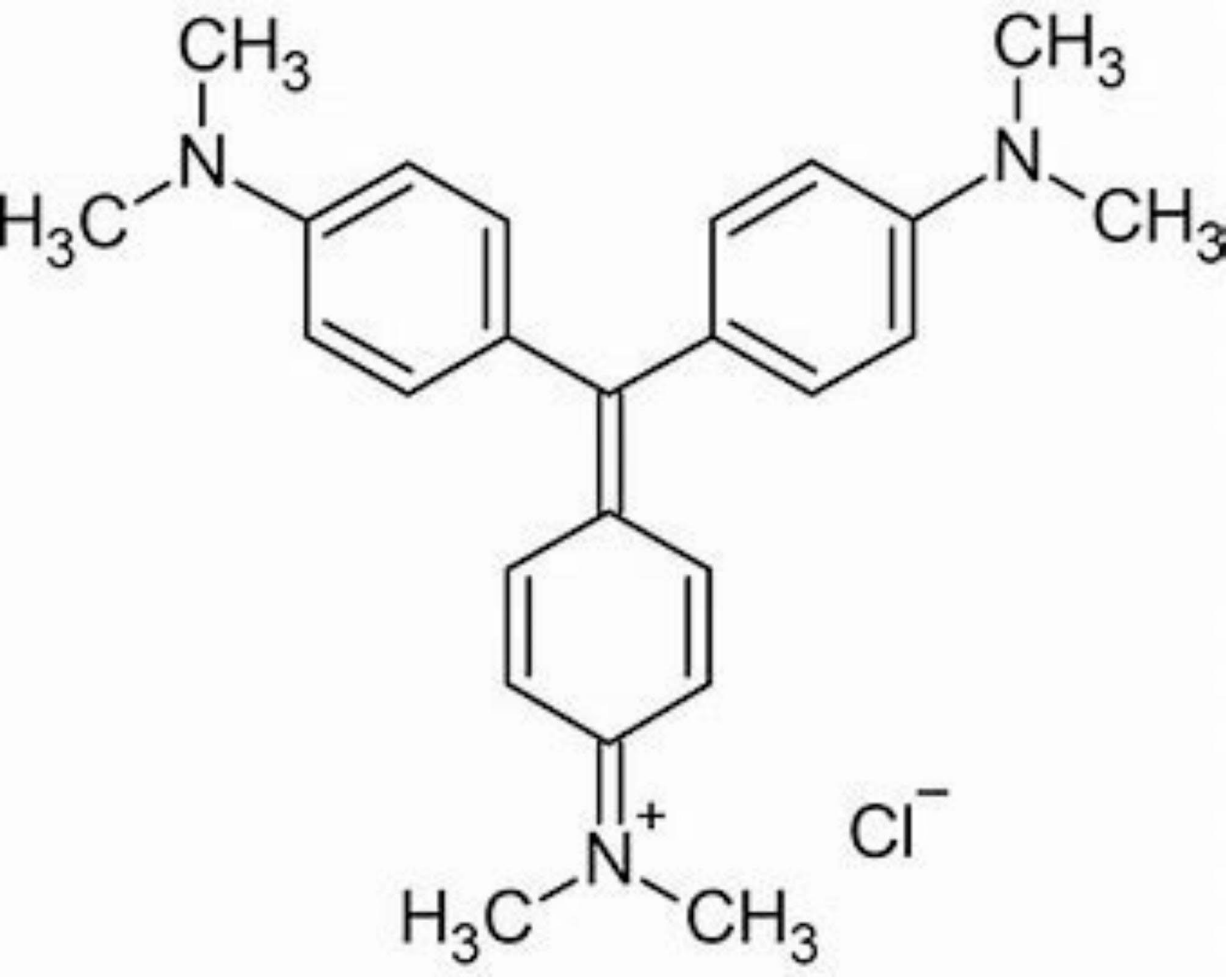
Structure of crystal violet.

Ground granulated blast furnace slag (GGBFS) with particles smaller than 100 μm and a Blaine surface area of 4700 × 50 cm^2^ per gram was supplied by Nile Oversees Company (Cairo, Egypt). Its oxide ratios (analyzed via XRF) are listed in Table 1. The fresh seeds of Moringa Oleifera (MS) were collected from the El Haden Area (El Behera, Egypt). Liquid sodium silicate (LSS) and sodium hydroxide (NaOH) flakes were provided by the Silica Egypt Company (Alexandria, Egypt), and EL Goumhouria Chemical Company (Cairo, Egypt). The silica modulus of SiO_2_/Na_2_O equals 2.80 and the liquid sodium silicate composition is 11.7 wt% Na_2_O, 32.8 wt% SiO_2_, and 55.5 wt% H_2_O. Crystal violet (molar weight = 408.0 g mol^–1^, purity of 99.0%, λ-max = 590) nm was bought from Prolabo company (France). Figure 1. displays the chemical structure of the CV. All the solutions were prepared using distilled water.

**Table 1:** Chemical oxide compositions (mass %) of slag.

Chemical composition of slag %										
CaO	Al_2_O_3_	SiO_2_	MgO	Fe_2_O_3_	SO_3_	K_2_O	Na_2_O	L.O.I	Cl^−^	H_2_O
42.56	7.02	32.86	11.58	1.14	2.50	0.15	0.29	0.93	0	0

**Fig. 2:**
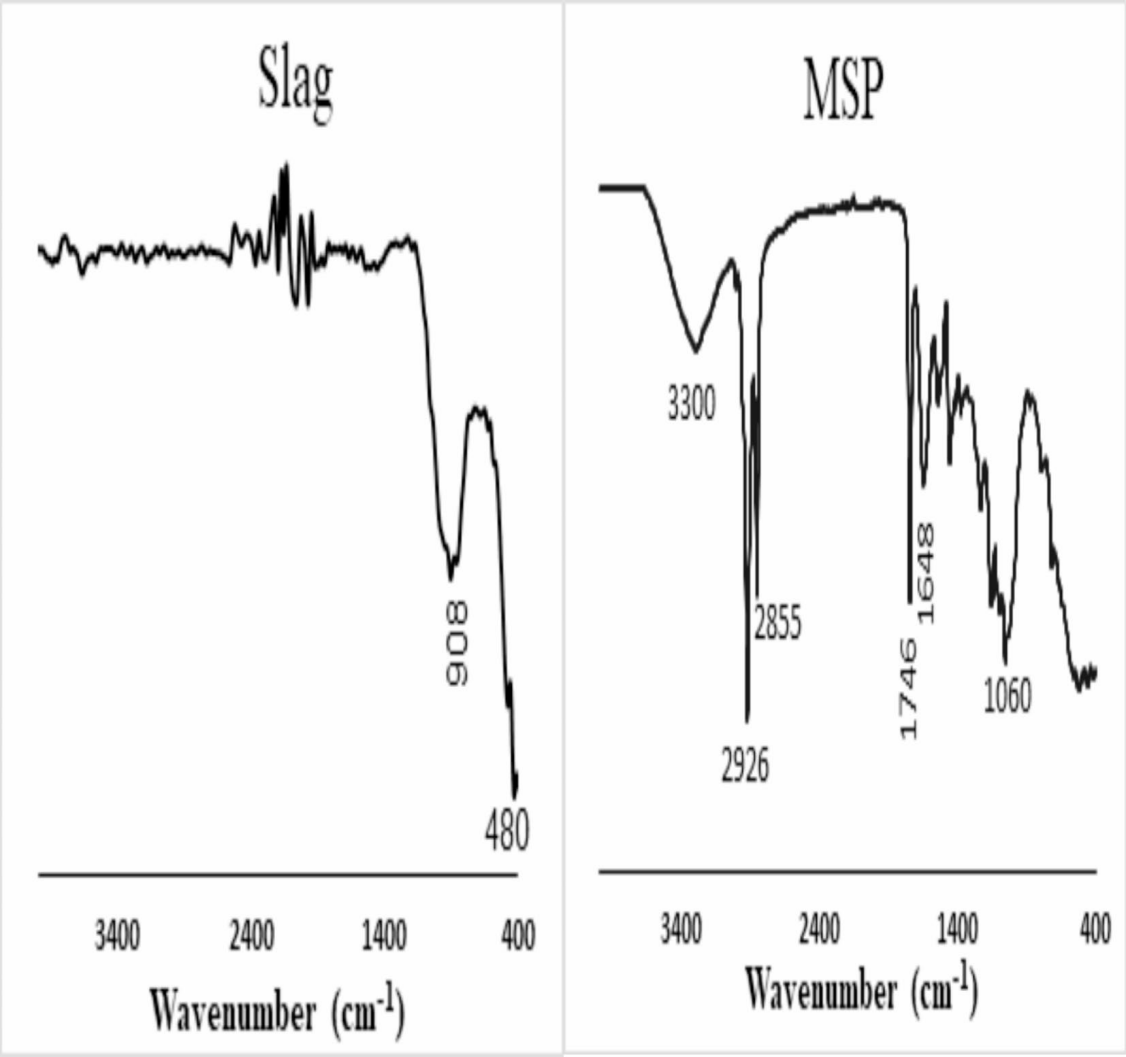
FTIR-spectra of ground granulated slag (GGBFS) and moringa seeds powder (MSP).

The FTIR of GGBFS reveals typical fingerprints of slag in the 980–1040 cm^− 1^ range linked to the asymmetric stretching vibration of Si–O–T (T = Si or Al) and 450–470 cm^− 1^, which indicates the Si–O–Si bending vibration mode’s presence (Fig. 2). The spectra Moringa seeds powder (MSP) Fig. 2. gives a wide band around 3300 cm^− 1^ linked to the O-H and N-H groups stretching band found in lignin, proteins, fatty acids, and carbohydrates. Strong peaks about 1746 cm^− 1^, caused by C = O stretching of the carbonyl groups in lignin and the acetyl ester in hemicellulose. The water absorbed in the material is linked to the strong broadband at 1,648 cm^− 1^ band. The C–O stretching is responsible for the peaks at 1,060 cm^− 1^.

### Methodology and characterization of geopolymer

#### Preparation of geopolymer

Moringa oleifera seeds are collected from agricultural zones. The outer husks or shells of the seeds were removed manually, and the seeds were cleaned with clean water to eliminate any dirt or impurities. The seeds were dried at 50^0^c for 24 h and then cooled at room temperature. A mechanical grinder was used to break the dried seeds into smaller pieces. An ultrafine grinder was used for fine grinding. The finely ground powder was passed through a 100-mesh sieve to ensure a uniform particle size distribution. The Moringa seed powder is Stored in a cool, dry place away from direct sunlight. Once sodium hydroxide (10 M) and sodium silicate powder were combined in a weight ratio of 2.5, the alkali activator solution was ready to be cooled for a full day. Using a liquid-to-solid ratio of 0.26, the dry mixes were combined with an alkaline activator solution for 15 min. After mixing, the sample pastes were placed into a 2.5 × 2.5 × 2.5-centimeter mold and cured for 24 h at 60 degrees Celsius. Demolded geopolymer cubes are kept at 100% humidity after the cure period. Table 2. shows the mix composition of the green-modified geopolymer cement pastes.

**Table 2:** The composition of the geopolymer paste mixes.

Mix code	S	SM1 (%)	SM2 (%)	SM3 (%)
Wt. % of slag	100%	99.8	99.4	99
Wt.% of moringa seed powder	0	0.2	0.6	1

#### Characterization of geopolymer

The compressive strength of each hardened paste is measured by Milano, Italy, D550-control type machine. After using an alcohol/acetone (1:1) solution to stop further hydration, the samples were dried for 24 h at 50 °C. The surface area analyzer known as Brunauer-Emmett-Teller (Quanta chrome model of NOVA touch 2LX, Milano-Italy machine) measures the overall porosity of every hardened paste. The phase composition, functional groups, and microstructure and morphological development of the hydration products were investigated using X-ray diffraction (Bruker D8 Discover diffractometer, Germany), Infrared spectrophotometer (PerkinElmer 1430 infrared spectrophotometer, USA), and High-resolution scanning electron microscopy (ESEM/Mapping: FEI, model Quanta FEG 250) respectively. MINI X, MICROTRAC was used to estimate the textural characteristics of S and SM3 using the adsorption-desorption of N_2_ gas at 77 K. Using the Brunauer-Emmett-Teller (BET) and Barrett-Joyner-Halenda (BJH) models, the specific surface area (m2/g), average pore diameter (nm), and total pore volume (cm3/g) were calculated. Using the pH drift approach, the study also involved calculating the point of zero charge (PZC) for each sample at ambient temperature^36^.

#### Adsorption test

The geopolymer cement samples used for batch adsorption (after 7 days) were squashed, ground, and screened to a particle size of 100 μm. It washed to attain pH 7, dried, and stored in a desiccator before their use. A 50 mL liquid was used for the adsorption testing, and 200 rpm of stirring speed was used. Dye stock solutions (1000 ppm) were prepared, and all the following experiments were done by diluting this solution. The adsorption capacity and removal efficiency at different pH (2, 4, 6, 8) were investigated and a selected pH range was used according to preliminary assays^37^. At the optimum pH, the adsorbent quantity (0.01, 0.03, 0.05, 0.07, 0.1, 0.15, and 0.2 g L^–1^), Time of contact (0, 30, 60, 120, 180, and 240 min), and dye concentration (10, 30, 60, 80, 100, 120 ppm) were investigated respectively to determine the optimum condition. The supernatant concentration was measured by UV/Visible spectrophotometer at λ-max = 590 nm. Dye removal efficiency (%) calculated by Eq. (1). Equations (2) and (3) were employed to determine the quantity absorbed at equilibrium (qe, mg g^–1^) and the amount adsorbed at a specific period (t) (qt, mg g^–1^), respectively.

1 $$\text{Efficiency} \left(\%\right) = \frac{C_{o}-C_{t}}{C_{o}} \times 100$$

2 $$\:\text{q}\text{e}=\frac{{C}_{o}\:-{C}_{t}\:}{\text{m}}\text{V}$$

3 $$\:\text{q}\text{t}=\frac{{C}_{o}\:-{C}_{t}\:}{\text{m}}\text{V}$$

Where V is the volume of solution (L), m is the quantity of adsorbent (g). The initial concentration of dye is denoted by C_0_ (mg L^–1^), and the concentration at time t is indicated by C_t_ (mg L^− 1^).

#### Isotherm models

Under the optimal conditions established from the adsorption test (Sect. "Adsorption test") for pH, adsorbent dose, and time, we investigated the adsorption behavior of varying dye concentrations (10, 30, 60, 80, 100, and 120 ppm) across the geopolymer composites (S, SM1, SM2, and SM3). Subsequently, we calculated the equilibrium concentrations (Ce) and the adsorption capacities (qe) for each geopolymer composite. The equilibrium data obtained for the adsorption of Crystal Violet (CV) onto the prepared geopolymer mixtures were analyzed using the Freundlich and Langmuir isotherm models. The linearized forms of the two isotherms are^38^ :

4 $$\frac{{{c_e}}}{{{q_e}}}=\frac{{{c_e}}}{{{q_m}}}+\frac{1}{{{K_L}{q_m}}}$$

5 $$\ln {q_e}=\ln {k_f}+\frac{1}{n}\ln {c_e}$$

The equilibrium concentration is C_e_ (mg/L), while the amount of dye adsorbed at equilibrium is q_e_ (mg/g). The Langmuir constant is K_L_ (L/mg) while the monolayer adsorption capacity is q_m_ (mg/g). The maximum CV absorbed into the geopolymer (q_m_) and the Langmuir constant K_L_ was derived from the chart of C_e_/q_e_ against C_e_, as shown in Eq. (4). The Freundlich constants, K_F_ and n, represent the adsorption intensity and capacity, respectively. Likewise, the plot of ln(q_e_) vs. ln (C_e_), as shown in Eq. (5), can be used to get the Freundlich isotherm constants K_F_ and n.

#### Kinetics models

In this study, we investigate the adsorption behavior of various geopolymer composites (S, SM1, SM2, and SM3) under optimal conditions, which include pH, adsorbent dose, and dye concentration, as detailed in Sect. "Adsorption test". The adsorption process is evaluated over different contact times: specifically at 0, 30, 60, 120, 180, and 240 min. This allows us to calculate the equilibrium concentration (Ce), the adsorption capacity at equilibrium (qe), and the adsorption capacity at time t (qt).The kinetics of adsorption are typically modeled using the pseudo-first-order and pseudo-second-order models. Ho and McKay formulated the pseudo-second-order model (Eq. 6)^39^, which demonstrates a linear relationship between t/qt and t. On the other hand, Lagergren described the pseudo-first-order model (Eq. 7)^38^, where a plot of log(qe − qt) against t yields a straight line.

6 $$\ln ({q_e} - {q_t})=\ln {q_e} - \frac{{{k_1}}}{{2.303}}t$$

7 $$\frac{t}{{{q_t}}}=\frac{1}{{{k_2}{q_e}^{2}}}+\frac{t}{{{q_e}}}$$

where the amount of CV adsorbed at equilibrium is denoted by qe (mg/g) and at time t by qt (mg/g). The pseudo-first-order adsorption rate constant is k_1_ (min ^− 1^) and the pseudo-second-order one is k_2_ (g mg ^− 1^ min ^− 1^).

## Result and discussion

### Mechanical characteristics

#### Compressive strength and porosity

**Fig. 3:**
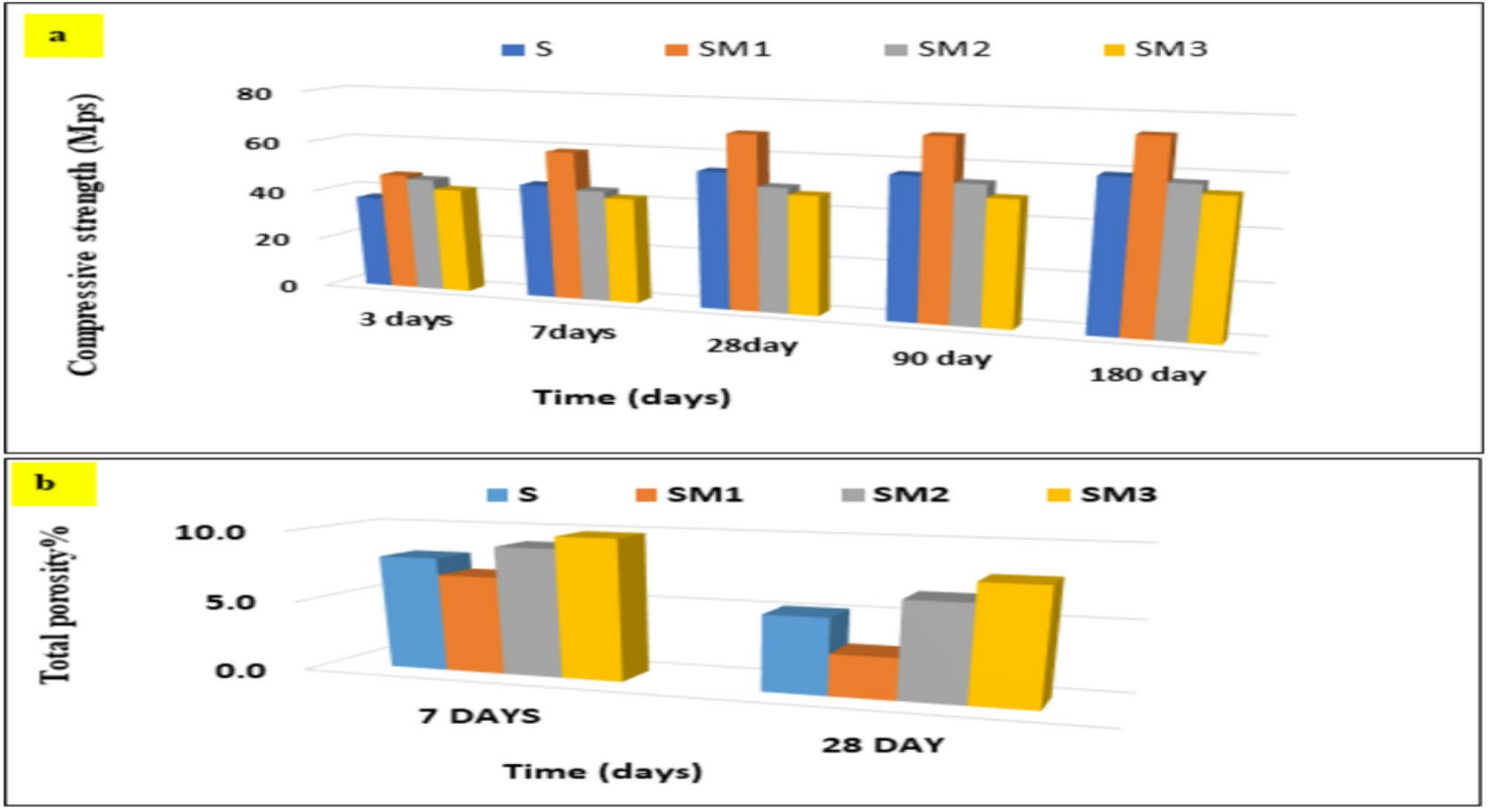
The values of (**a**) compressive strength and (**b**) total Porosity % for the geopolymer composites at different hydration ages.

The compressive strength and total porosity of the control specimen and modified geopolymer with 0.2, 0.6, and 1 wt% MSP (S, SM1, SM2, and SM3 respectively) cured up to 180 days are displayed in Fig. 3. A significant increase in the compressive strength is obtained in all mixes over time because of the progress of the geopolymerization reaction that leads to the growth of more phases that provide strength (Both calcium-alumino-silicate-hydrate (C-A-S-H) and calcium-silicate-hydrate (C-S-H)) in open pores decreasing the porosity and creating a composite that is more compact and has strong mechanical properties^40^. Adding 0.2 wt% MSP (SM1) gives compressive strengths higher than the control mix, probably due to (i) MSP acting as filler, sealing the microcracks, capillary holes, and voids to form a compact structure, and (ii) The creation of a network structure with organic-inorganic cross-links and activation ability of MSP^41^. Increasing the percentage of MSP decreases compressive strength value as shown in SM2 and SM3. As the content of Moringa seed powder increases, the chances of particle agglomeration also go up. When particles stick together, they create larger aggregates instead of spreading evenly throughout the mixture. This agglomeration can hinder proper bonding between the Moringa powder and other materials for efficient polymerization or hydration processes that support strength development. In addition, the agglomerated Moringa particles can act as stress concentrators when mechanical loads are applied. These clusters may not distribute the stress evenly across the material, resulting in localized failures or microcracks that can spread through the structure and further diminish compressive strength^42,43^. These results are supported by XRD, FTIR, and SEM.

#### FTIR spectrum

**Fig. 4:**
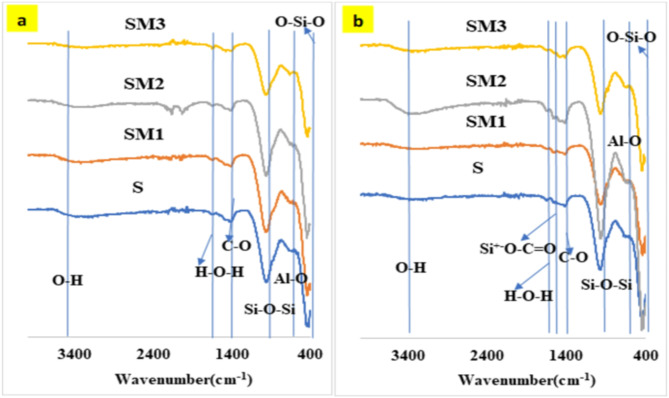
FTIR-spectra of the geopolymer composites at (**a**) 7 days and (**b**) 180 days.

The Fourier transform infrared (FTIR) analysis of the control and three slag/moringa seeds powder geopolymer pastes, including S, SM1, SM2, and SM3, after (7) and (180) days of hydration can be detected in Fig. 4. We can observe Two absorption bands in the FTIR spectrum located at 3400 –3200 cm^− 1^ and 1644–1649 cm^− 1^ because of the vibration modes of the O-H group stretching and the H-O-H group bending, respectively^44^. The band at 1420–1450 and 870–875 cm^− 1^ is due to the C-O bond’s stretching vibration in CO_3_ ^− 2^ groups, which is formed during the carbonation process of Ca (OH)_2_. The band in the 1000–950 cm^–1^ range was connected to Si-O-T’s asymmetric stretching vibration (where T can be either Si or Al). A slight shift to a lower wave number in the (Si–O–T) asymmetric stretching vibration band after 180 days confirms amorphous aluminosilicate gels (C-S-H and C-A-S-H) produced by the geopolymerization process in geopolymer binders^45^. According to Fig. (4b), the appearance of a new band at 1554 cm^–1^ after 180 days in SM1 is related to the carboxylate group (arising from the interaction between the carbonyl group in moringa seeds and the hydroxyl groups on the geopolymer surface (Si^+−^O-C = O)^46^. These results are attributed to the positive effect of 0.2%wt MSP in the creation of an organic-inorganic cross-linking network structure in the geopolymer matrix, filling the open pores and are in good agreement with the mechanical results. Comparing the SM1 mix to other mixes including a greater amount of MSP, there is a higher intensity of the asymmetric stretching vibration of the Si-O-T band at 965 cm^–1^., which causes the drop in the geopolymerization rate owing to agglomeration of MSP which coats the unreacted slag particles as well as hydration products. Unreacted slag was linked to the Si-O-Si bending vibration mode that occurred at about (448–415) cm^–1^^46^.

#### Analysis of X-ray diffraction

**Fig. 5:**
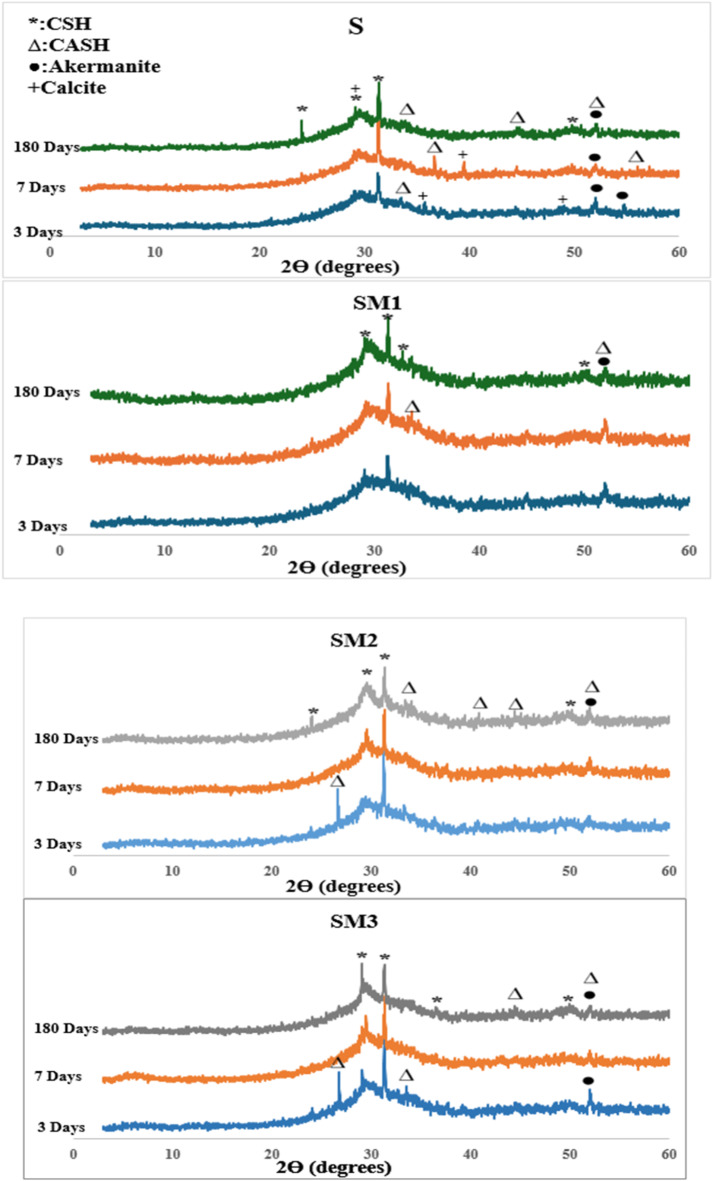
XRD of the geopolymer composites at 3, 7, and 180 days.

The XRD patterns of the control mix (S) and the modified geopolymer (SM1, SM2, and SM3) after 3,7 and 180 days are displayed in Fig. 5. Due to the dissolving of slag by alkaline activation and the development of an amorphous geopolymer matrix, all geopolymer samples exhibited a significant and broad hump in their XRD patterns between 20° and 40° 2θ. In XRD patterns the presence 2θ = 24, 31, 44 indicates the development of a newly crystalline phase, possibly a gel of type (CASH, PDF# 00–002–0047) at d-values of 2.8, 2.4, 2.08 and 1.6 Å, resulting from the substitution of free silica with aluminum (Al) atoms during the geopolymerization process^47^. The crystalline phase (CSH, PDF# 00–002–0068), exhibiting basal reflections at d = 3.5, 2.9, 2.78, and 1.8 Aº at 2θ = 24. 31, 32, and 50° respectively were formed during the geopolymerization process. This implied that both amorphous and crystalline phases were formed as a result of the geopolymerization process. The appearance of calcite (PDF# 00-047-1743) peaks at d = 3.8, 2.4, 2.2, 1.8 A^o^ at different curing time up to 180 days mainly because of carbonation. The presence of MSP did not affect the characteristics of the hydration product present in the samples, as demonstrated in Fig. 5. In SM1 the presence of 0.2%wt MSP in the geopolymer mix increases the strength of the distinctive peaks of the hydration product (CSH, CASH). On the other hand, SM2 and SM3 exhibited a reduction in CSH peak. This is in good agreement with the compressive strength results.

#### SEM analysis

**Fig. 6:**
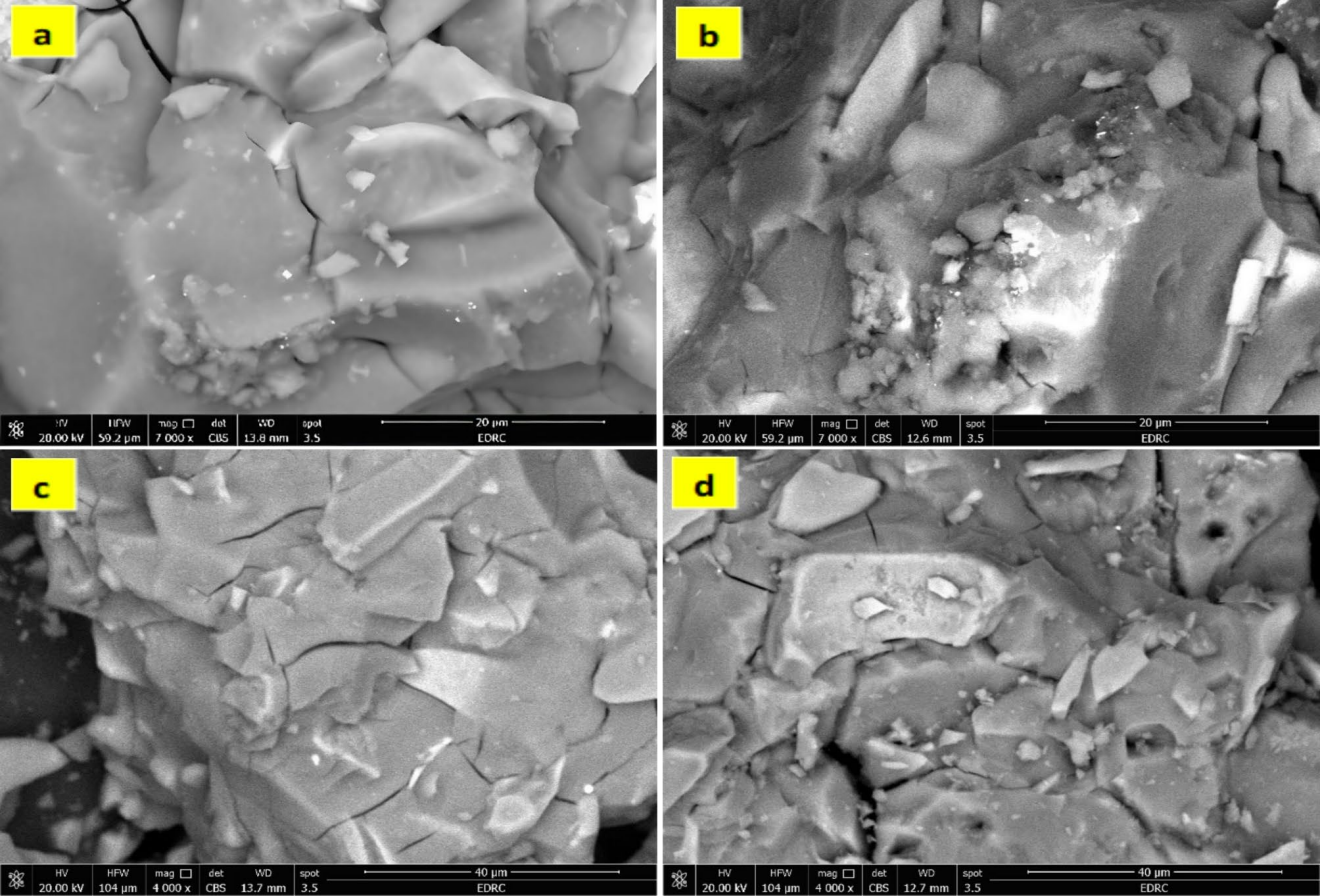
SEM micrographs of hardened geopolymers composites: (**a**) S, (**b**) SM1, (**c**) SM2, and (**d**) SM3 before the adsorption of CV.

Figure 6. shows the microstructures of the control mix (S) and the modified geopolymer (SM1, SM2, and SM3) after 7 days of curing. SEM analysis (Fig. 6a) reveals a uniform, amorphous structure signifying the effective completion of the geopolymerization process with the appearance of a small number of microcracks on the surface of the specimens. This crack is mainly due to the nonuniform shrinkage in the geopolymers or excessive water evaporation during the hardening process^48,49^. Using 0.2%wt MSP gives a more compact structure with fewer microcracks. On the other hand, Increasing the amount of MSP increases the heterogeneity of the matrix, the addition of 1%wt MSP (SM3) appears to be less compact, with excess microcracks and big particles dispersed over the matrix of the geopolymer, that could be explained by the accumulation of MSP particles^50^. This observation was in good adherence to mechanical properties results, which give lower mechanical properties than other mixes.

#### Pore structure analysis

**Fig. 7:**
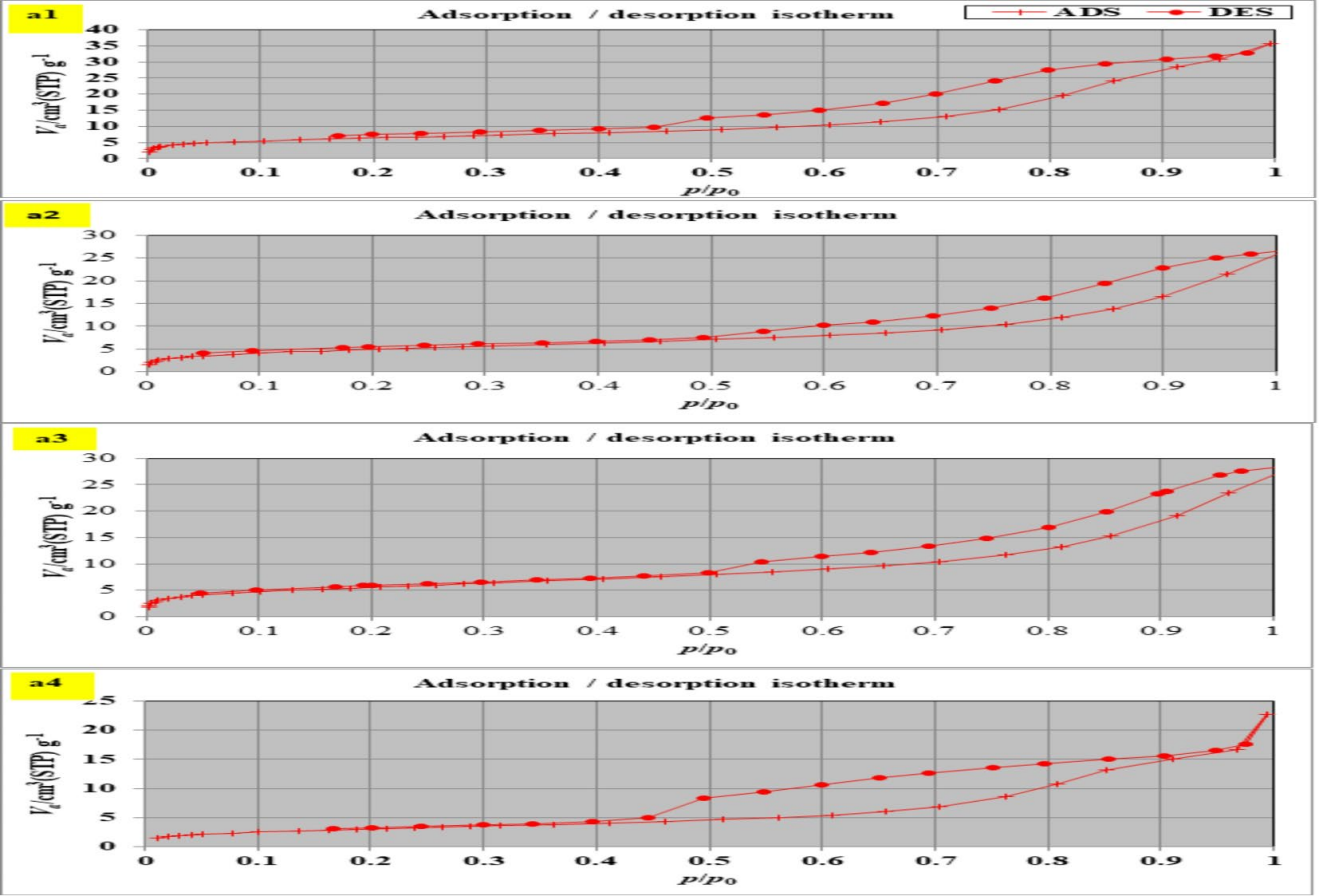

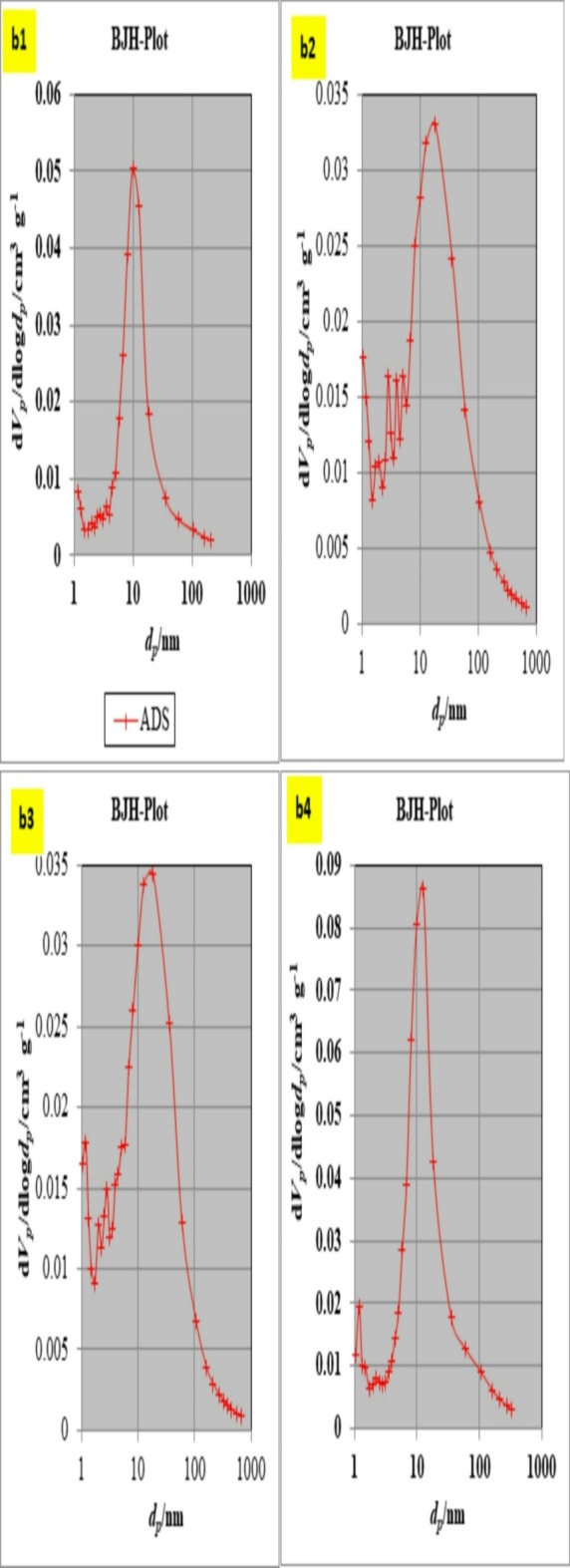
N_2_-adsorption/desorption isotherm for (**a1**) S, (**a2**) SM1, (**a3**) SM2, and (**a4**) SM3, BJH- pore size distribution for (**b1**) S, (**b2**) SM1, (**b3**) SM2, and (**b4**) SM3.

The N_2_ adsorption isotherms for the S, SM1,SM2, and SM3 can be categorized by the IUPAC as type IV isotherms with an H3 hysteresis loop (Fig. 7a)^51,52^. The data from Table 3. show that increasing the percentage of moringa seeds increases surface area and the presence of 1% moringa seed powder effectively enhances the surface area and pore volume of modified geopolymer whereas, the average pore radius decreases giving high adsorption capacity^53^. The pore size distribution plot shows that the synthesized geopolymer materials are mostly microporous (Fig. 7b). Adding moringa seed to the mix resulted in a material with enhanced mesoporosity BJH-MPD.

**Table 3:** The main parameters for pore structure characteristics in various geopolymer composites before and after adsorption.

Adsorbent	SBET, m^2^.g^− 1^	Volume of the pores, cc.g^− 1^	Average pore radius, nm
Before CV adsorption			
S	11.277	0.034	6.023
SM1	17.52	0.038	4.379
SM2	19.882	0.0402	4.044
SM3	22.709	0.054	4.765
After CV adsorption			
S	9.295	0.022	4.859
SM1	10.54	0.025	4.023
SM2	16.45	0.038	4.019
SM3	18.78	0.040	4.001

### Adsorption investigations

The study examines the impact of incorporating agricultural waste (MSP) into slag, an industrial by-product, for the treatment of wastewater containing CV dye via an adsorption method. This approach functions both as a treatment process and as an ingredient in building materials.

#### Impact of pH

**Fig. 8:**
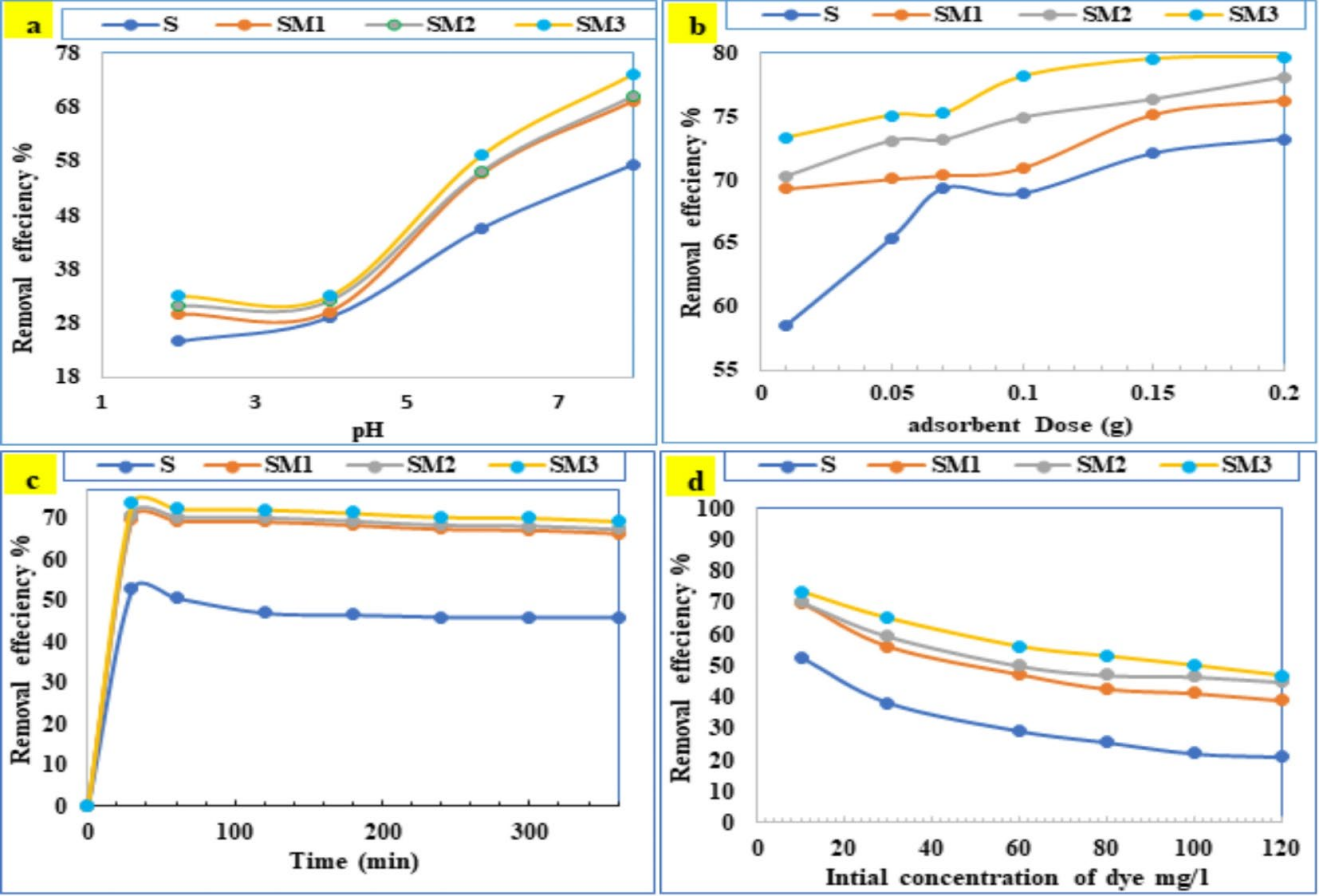
The effect of (**a**) solution pH, (**b**) adsorbent dose, (**c**) contact time, and (**d**) initial concentration of the crystal violet dye, on the dye removal efficiency using different geopolymer mixes.

**Fig. 9:**
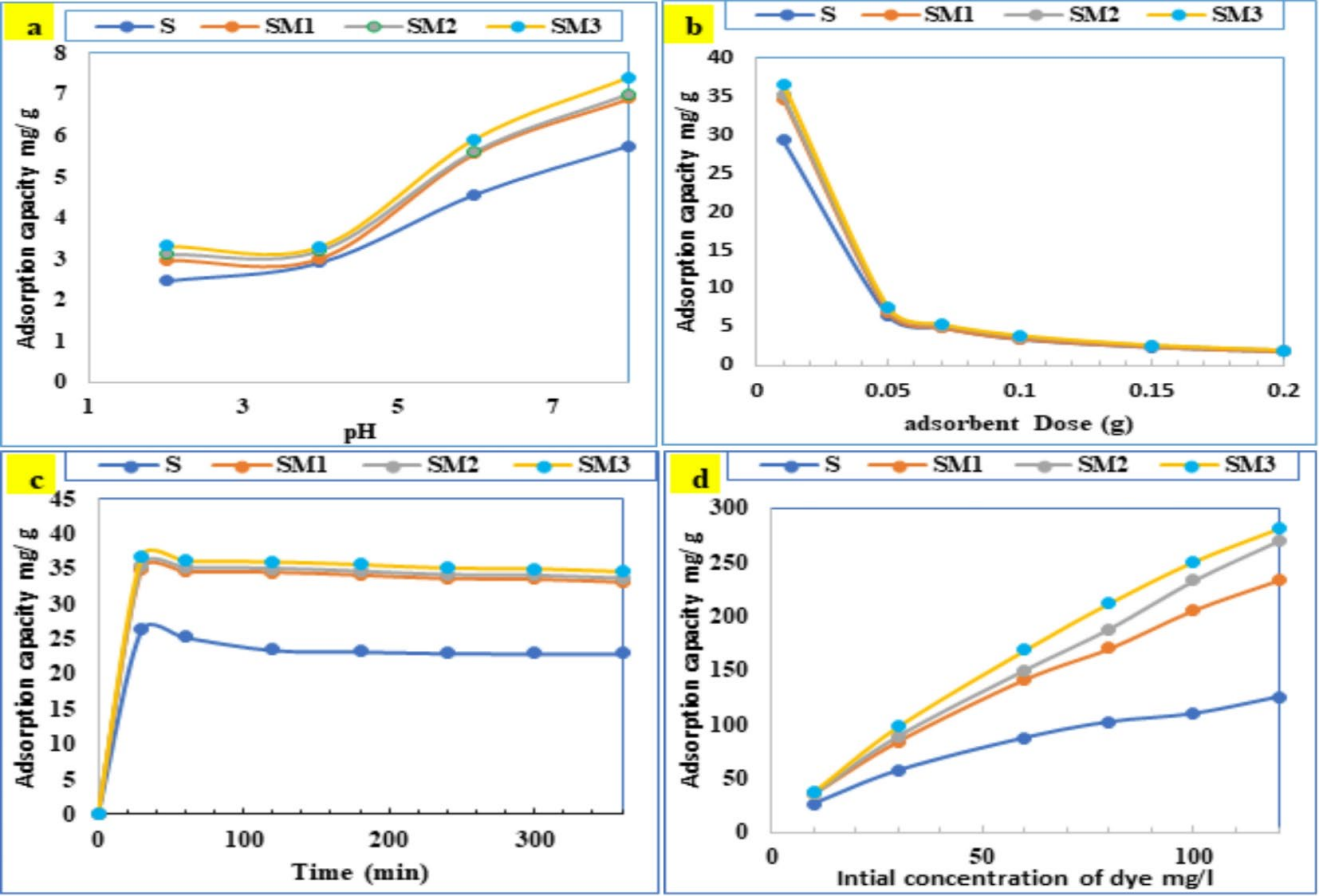
The effect of (**a**) solution pH, (**b**) adsorbent dose, (**c**) contact time, and (**d**) initial concentration of the crystal violet dye, on the dye adsorption capacity using different geopolymer mixes.

The valuation of the effectiveness of adsorption of the mixes was started by optimizing the solution’s pH because the pH variation alters the adsorbent charge of the surface through interactions with functional groups on the surface-active sites. The pH above 8 is not considered because, in a highly alkaline medium (pH > 8), the cationic form can lose protons, resulting in neutral or anionic species. These forms are less soluble in water and may precipitate^37,54^. The removal efficiency and adsorption capacity of mixes at different pH ranges (2–8) are shown in Figs. 8 and 9. These forms are less soluble in water and may precipitate. The prepared geopolymer exhibited PZC around pH 8 as shown in Table 4. It is found that both adsorption capacity and removal efficiency increase with increasing pH. This performance is possibly related to how the geopolymer function groups (siloxane groups) modify in acidic and basic solutions^55^. At pH 2.0, lower than PZC, the protonation of siloxane groups hugely reduces the CV elimination because of electrostatic repulsive forces with the positively charged geopolymer sites. Between pH 4.0 and 6.0, there was a slight increase in CV removal because of the geopolymer active site’s deprotonation^56^. At pH 8.0, the adsorption process was significant because of electrostatic interactions between the negatively charged geopolymer sites and the positively charged CV. Furthermore, a significant factor contributing to the increased dye removal was the hydrogen bond between the CV dye molecule and the –OH groups on the geopolymer surface^57^. The optimum pH for CV adsorption on the geopolymer surface was 8, and this value was chosen for further optimization.

**Table 4:** Point of zero charge of prepared geopolymer.

Geopolymer	S	SM1	SM2	SM3
PZC	8.7	8.25	8.35	8.3

#### Effect of adsorbent dose

The effect of the adsorbent amount (0.01 g to 0.2 g) on the CV adsorption capacity for adsorption and elimination efficiency by the geopolymer was estimated in Figs. 8 and 9. The CV adsorption capacity and the removal efficiency were changed inversely by the rise in the geopolymer dose, the adsorption capacity decreases, and the removal efficiency raises when the geopolymer dose increases. By increasing the amount of adsorbent, more adsorption sites become available, raising the quantity of CV that is adsorbed. Conversely, the quantity adsorbed per unit of mass (i.e. adsorption capacity) drops which is illustrated by the existence of unsaturated adsorption sites^58^. Besides, The surface area decreases due to the increasing adsorbent aggregation^23^. The drop in the CV adsorption capacity when mounting the adsorbent dose has been formerly reported^59^. 0.01 g was taken as the optimum dose as it gives high adsorption capacity.

#### Effect of contact time

Figures 8 and 9 displays the impact of the contact time on CV elimination using a different contact time of 30 to 360 min. The CV initial concentration was 10 mg/L with an optimum geopolymer dosage of 0.01 g/L and pH 8. It shows that high CV removal occurs in the first 30 min, with removal efficiency ranging from 52 to 86%. In contrast, adsorption capacity ranges from 26 to 43% depending on the composition of the geopolymer mixes, while afterward a gentler removal rate is observed up to 360 min. For all geopolymer mixes both the adsorption capacity and removal efficiency increase as the contact time increases till they reach equilibrium at 30 min. While longer sorption times did not induce significant gains, so 30 min can be considered the equilibrium time. This is conceivably owing to the adsorbent’s large surface area particles readily available early in the adsorption process and after a specific amount of time the active sites become fully occupied^57^.

#### Effect of dye concentration

Figures 8 and 9 represents the impact of different initial concentrations of CV (10 to 120 mg/L) on the sorption effectiveness and capacity of the geopolymer sample. It is seen that the removal efficiency shows high values at lower initial concentration and then decreases as the initial concentration increases from 10 mg/L. The reason might be due to the saturation of the geopolymer’s active sites, which interact with CV^60^. The adsorption capacity of CV increases as the initial CV concentration increases, owing to the rise in the driving force of the concentration gradient that encourages more dye molecules to diffuse from the solution toward the geopolymer surface^57,61^.

Our results indicate that combining slag and Moringa seeds shows promising potential for removing crystal violet dye from wastewater. This innovative method not only helps the environment but also offers economic benefits for wastewater treatment. By using these eco-friendly geopolymers, industries can effectively address dye pollution while also lowering costs compared to traditional treatment methods. Additionally, incorporating industrial and agricultural waste into wastewater treatment promotes sustainability by minimizing the amount of waste sent to landfills.

#### Adsorption kinetics

The adsorption kinetic of the CV onto geopolymer was investigated by two models within 360 min, pseudo-first-order Eq. (4) and pseudo-second-order Eq. (5)^62^. The experimental kinetic data for the samples was calculated and summarized in Table 5. The pseudo-second-order model’s correlation coefficient (R^2^) obtained from the samples reached the highest value, which reveals that the absorption process of CV onto the modified geopolymer samples follows the pseudo-second-order kinetic model.

**Table 5:** Adsorption’s kinetic parameters of various modified geopolymer cement mixes towards crystal Violet dye.

Kinetic parameter					
Type of model	Parameters	S	SM1	SM2	SM3
Pseudo-first-order	q_e_ (mg/g)	0.12536	1.7622	2.4164	1.2688
	K_1_ (L/mg)	0.000003	0.00001	0.000004	0.000008
	R^2^	0.6306	0.9336	0.9746	0.8887
Pseudo-second-order	q_e_	22.5734	33.0033	33.557	34.6030
	K_2_	−0.0043	−0.00985	−0.00728	−0.00743
	R^2^	0.9999	0.9998	0.9998	0.9998

#### Adsorption isotherm

The adsorption isotherm is a significant factor as it shows the ways in which the adsorbent and adsorbate interact. The equilibrium adsorption isotherms of crystal violet using the modified geopolymer as adsorbent are accomplished using Langmuir and Freundlich models. Langmuir isotherm model supposes monolayer adsorption because of the regular adsorptive surface. Equation (6), The Freundlich isotherm model accounts for multilayer adsorption due to the heterogeneous character of the adsorbent surfaces, which have different adsorption capacities^63^. Table 6 displays the isotherm parameters and coefficients of squared correlation (R^2^) for each isotherm model. The Freundlich model is the best fit for the experimental data across all mixes, as it exhibits higher R² values compared to the Langmuir model. This indicates a strong correlation between experimental and predicted values. The n values for S, SM1, SM2, and SM3 are greater than 1 and smaller than 10, indicating favorable adsorption of dyes onto the adsorbent surface^64^. The Freundlich model’s ability to describe multilayer adsorption further supports its suitability for this study according to the results^58^. Additionally, K_F_ values for S, SM1, SM2, and SM3 are 12.20, 18.20, 17.38, and 20.49, respectively, demonstrating that K_F_ > 1. This result indicates that the Freundlich theory is supported and enhanced by the adsorption interaction process on all modified geopolymer mixes. The R_L_ values further confirm that the adsorption of CV onto the geopolymer is favorable. Lastly, the variations in K_L_ values between different mixes highlight differences in dye binding strength and capacity with the geopolymer’s surface^58^.

**Table 6:** Isotherm parameters for the adsorption behavior of various modified geopolymer cement mixes towards crystal Violet dye.

Isotherm parameter					
Type of isotherm	Parameters	S	SM1	SM2	SM3
Langmuir	q_max_ (mg/g)	131.5789	227.2727	277.7778	322.5806
	K_L_ (L/mg)	0.052234	0.05954	0.047745	0.048287
	R_L_	0.656885	0.626802	0.67684	0.67437
	R^2^	0.9908	0.9865	0.9905	0.996
Freundlich	K_F_	12.198319	18.197009	17.378008	20.492739
	n	1.9395	1.6900	1.5069	1.5601
	R^2^	0.996	0.9959	0.9982	0.9977

#### Morphology of geopolymer composites after crystal violet adsorption

To understand the mechanism of CV adsorption on geopolymer composite several analyses such as XRD, SEM, FTIR, and pore size analysis were conducted.

##### XRD analysis

**Fig. 10:**
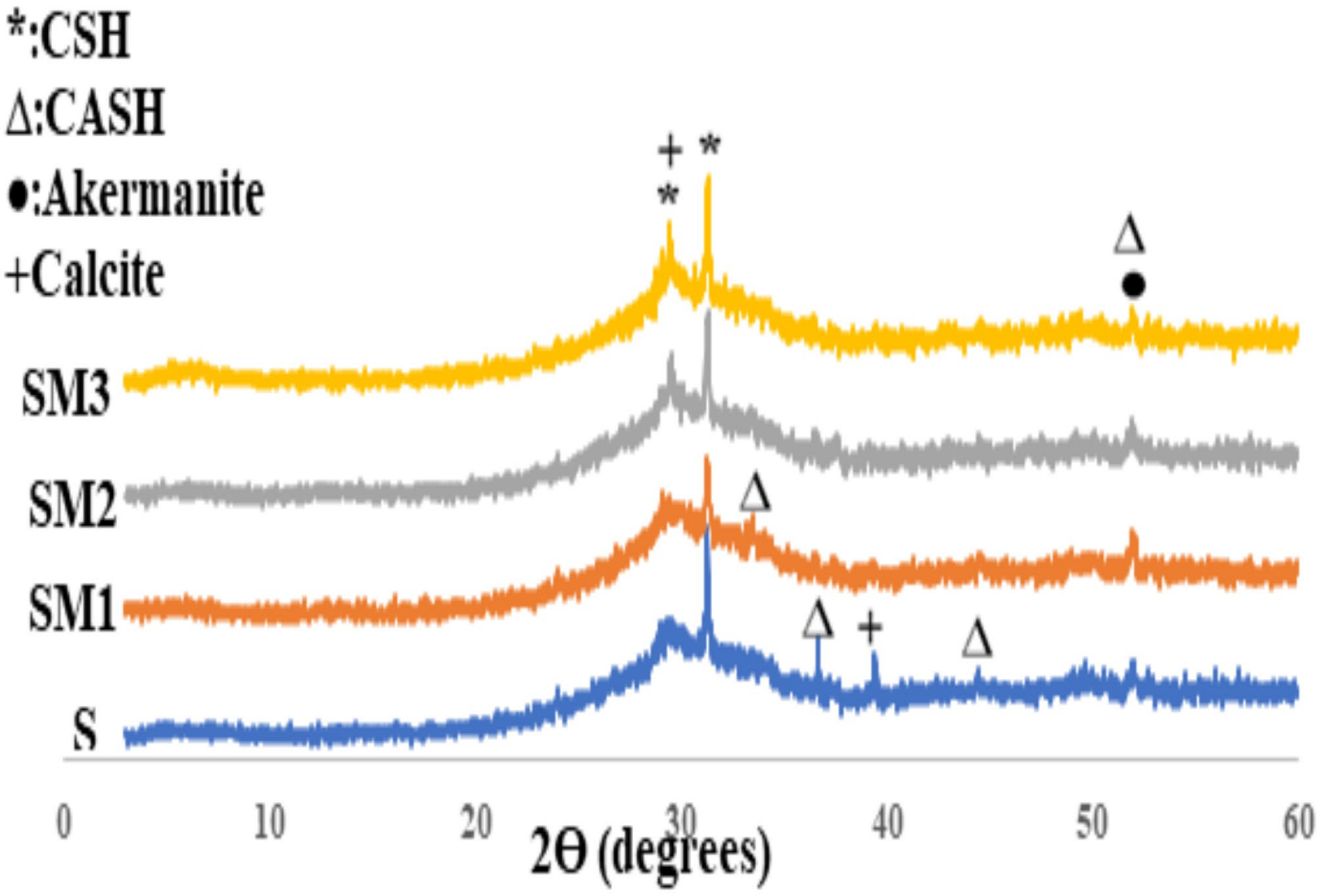
XRD of hardened geopolymer composites S, SM1, SM2, and SM3 after the adsorption of CV.

Figure 10 shows the XRD of geopolymer samples after CV adsorption. We can observe that there were no major changes in peak locations and peak intensity after CV adsorption^65^. To investigate this result further, SEM, FTIR and pore size analysis were carried out on all samples.

##### SEM analysis

**Fig. 11:**
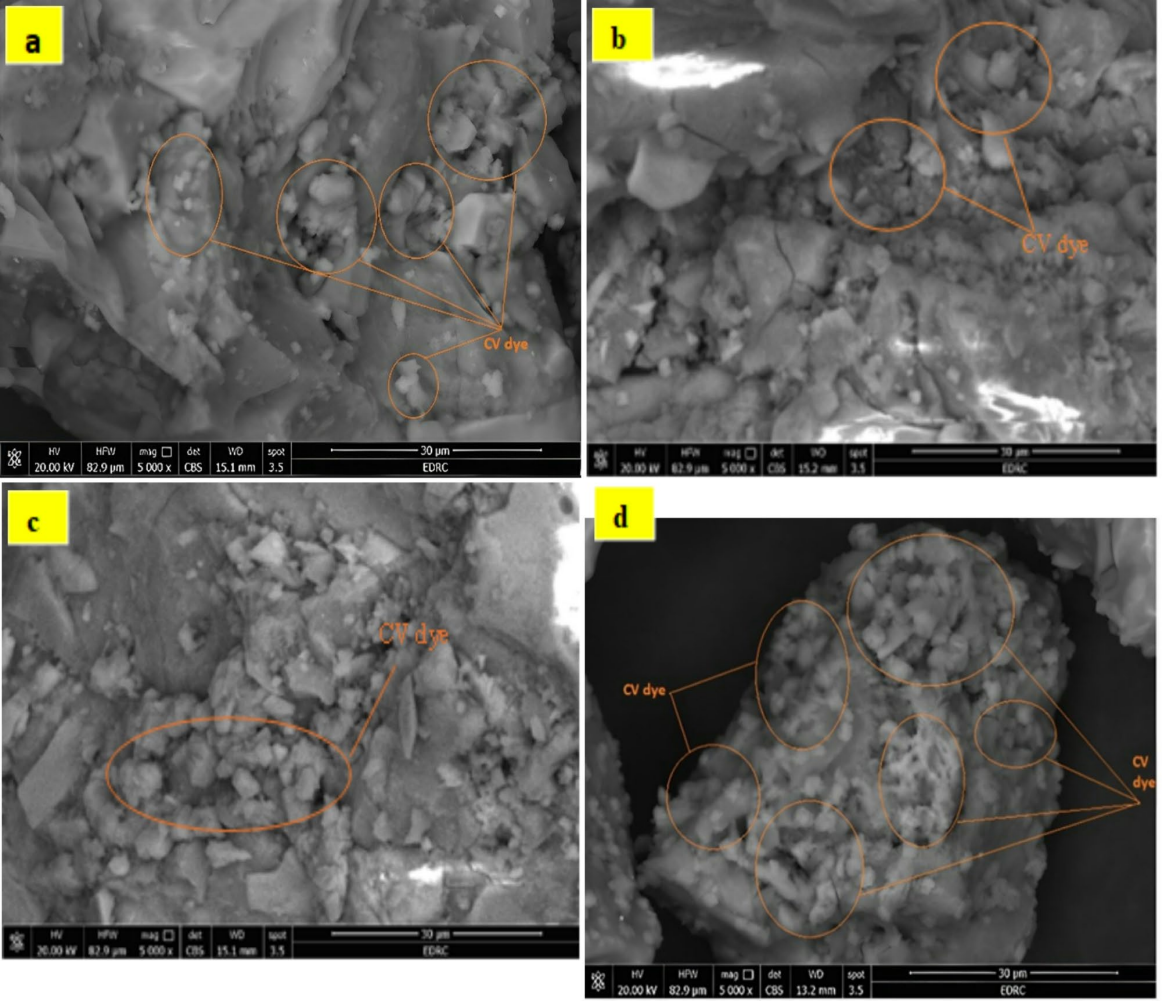
SEM micrographs of hardened geopolymer composites: (**a**) S, (**b**) SM1, (**c**) SM2(**d**), and SM3 after the adsorption of CV.

Figure 11a–d. represents the microstructure of S and SM1, SM2, and SM3 after absorbing crystal violet dye. A thin, uniform layer of Crystal Violet dye covers the surface, reducing the visibility of pores, indicating dye molecules have filled or blocked them. Comparing the specimens, SM3 shows a fully covered surface than other mixes and this is clearly because of the heterogeneous morphology of SM3 with cracks (Fig. 7a) in addition to the protein component of the MSP which facilitates the processes of CV adsorption^66,67^. Accordingly, based on SEM images, it can be deduced that the modified geopolymer morphology is suitable for dye adsorption.

##### FTIR analysis

**Fig. 12:**
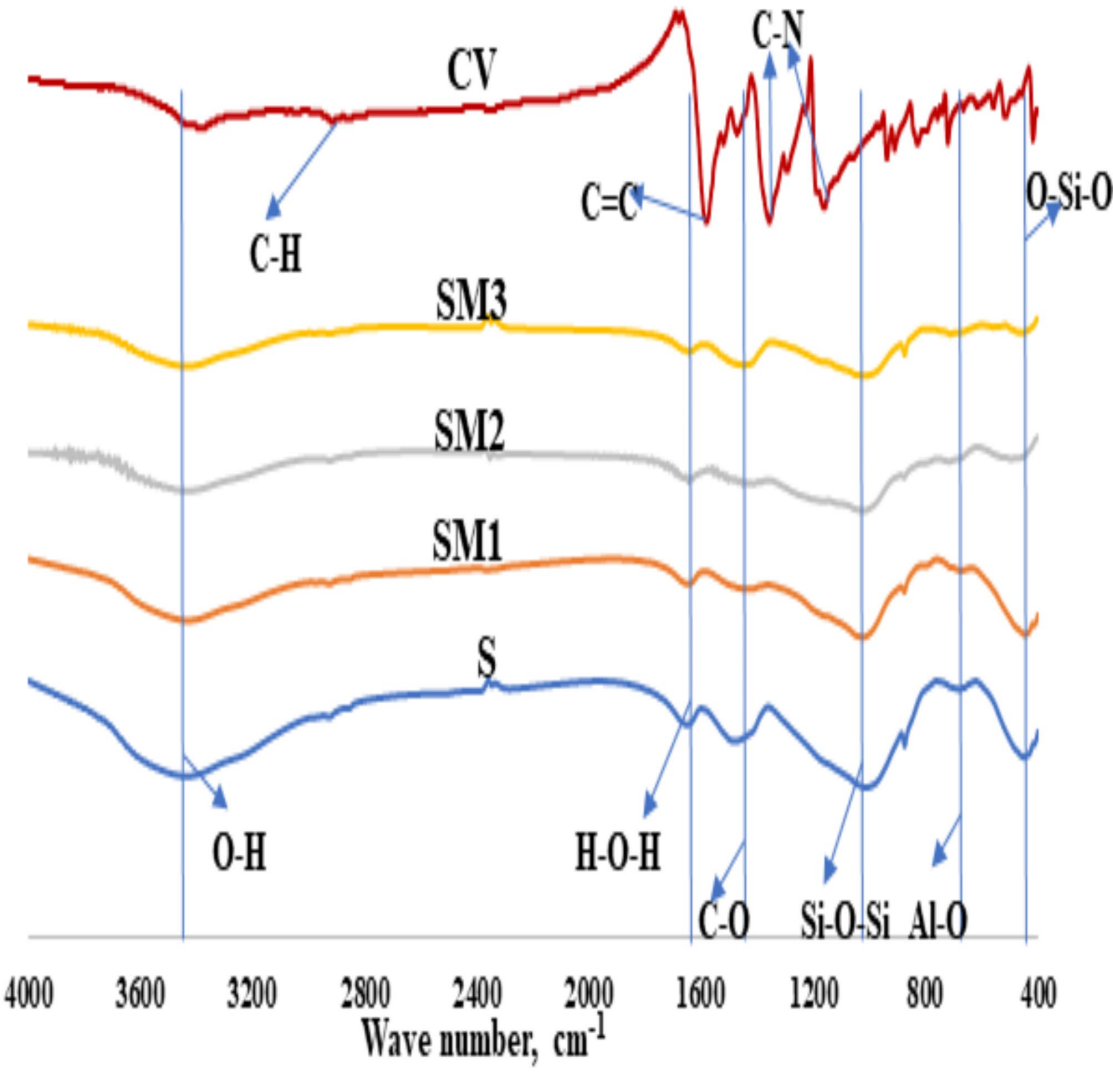
FTIR-spectra of the geopolymer composites after adsorption.

Figure 12 shows the Fourier Transform Infrared (FTIR) spectra of crystal violet and slag geopolymer composites after adsorption. Crystal violet exhibits characteristic bands associated with its functional groups, including a weak aromatic C-H stretching band around 2921 cm^− 1^, a C–N stretching Vibrations band at 1161 cm^− 1^, a C = C stretching band of aromatic benzene ring at 1581 cm^− 1^, a C-N stretching band of aromatic tertiary amine between 1357 cm^− 1^, and a weak band in at 2369 cm^− 1^corresponding to a symmetric and asymmetric stretching of tertiary amine salt^68^. After adsorption, there is clear reduction and shift in the asymmetric stretching vibrations of Si-O-T and the O-H stretching bands. This behavior is most likely attributable to the ability of crystal violet to be easily absorbed into the geopolymer composite.

##### Pore size analysis

**Fig. 13:**
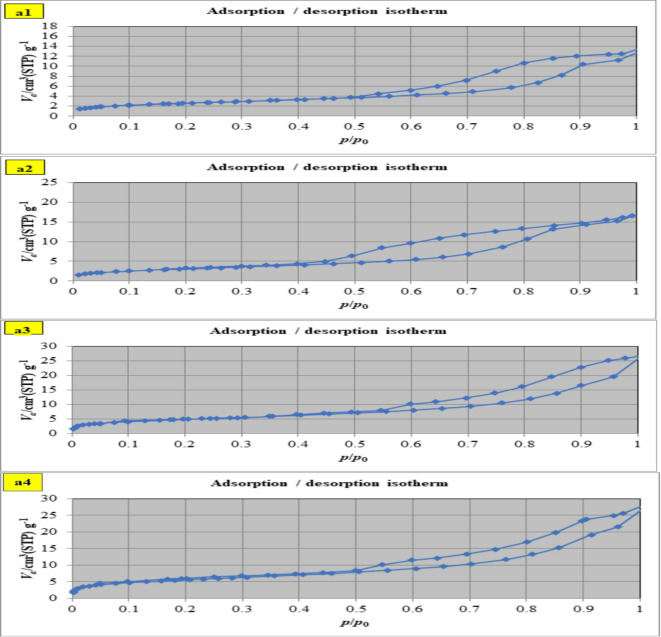
N_2_-adsorption/desorption isotherm for (**a1**) S, (**a2**) SM1, (**a3**) SM2, and (**a4**) SM3 after CV adsorption.

The surface properties of the control (S) and the modified geopolymer mixes (SM1, SM2, and SM3) after adsorption were characterized by nitrogen adsorption − desorption measurement (Table 3; Fig. 13). There is a clear reduction in values of surface area, average pore diameter, and total pore volume which confirms the higher adsorption properties of prepared geopolymer composite^69^.

#### Adsorption mechanism and comparison with other adsorbents in different studies

**Fig. 14:**
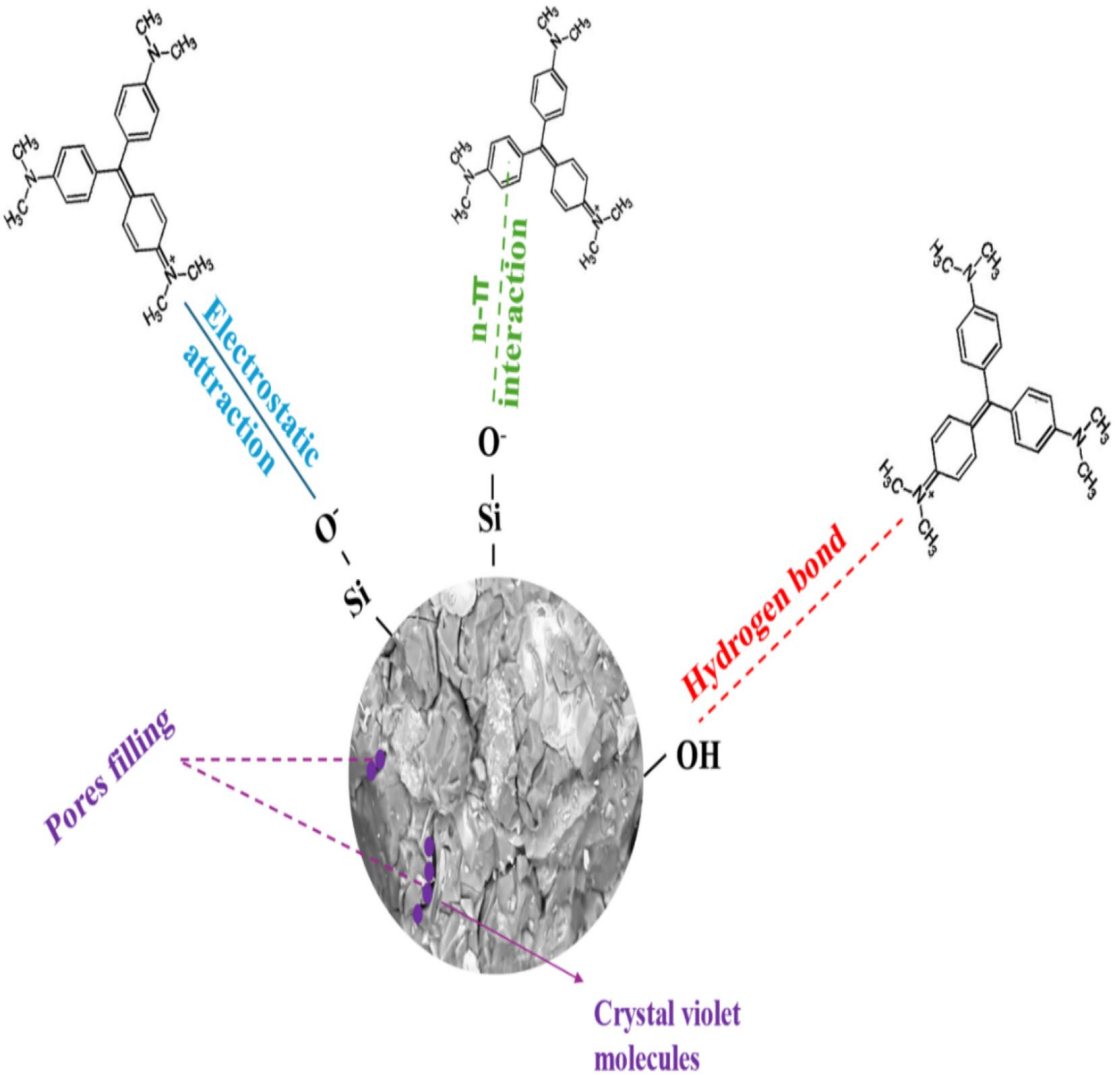
Adsorption mechanism proposed for geopolymer adsorbent composites.

The cationic CV dye’s retention mechanism on geopolymer composite is complex, with several interactions between the dye molecules and the adsorbent surface including^70,71^: Hydrogen bonding between the nitrogen atoms (N^+^) in CV dye molecules with the hydroxyl groups (OH^−^) of the geopolymer matrix. Kinetic studies, including the pseudo-second order model, indicate that chemisorption involves ionic interactions between dye molecules and geopolymers^72^. Electrostatic attraction between the geopolymer adsorbent with a negatively charged surface and positively charged dye molecules at pH 8. Dye adsorption also relies heavily on the porous structure of geopolymer, as proven by BET/BJH surface and pore studies. The micro- and mesopores hold CV molecules. These mechanisms describe the complicated adsorption of CV dye onto geopolymer adsorbents, supported by both experimental and theoretical data.

considerations, which are depicted graphically in Fig. 14. Our study also indicates that the adsorption of CV dye molecules may occur through n-pi interactions between their aromatic structures and the active Si-O-Si/Al sites in geopolymers^73^. Chemisorption, driven by valence forces from electron sharing or exchanging between geopolymer composites and dyes, plays a crucial role in this process. This strong interaction results in high removal efficiency of CV dye molecules from wastewater, positioning geopolymer composites (S, SM1, SM2, SM3) as promising materials for environmental remediation. However, further research is necessary to enhance the reusability of these geopolymer composites for CV dye removal and to facilitate their application in actual industrial water treatment processes.

Table 7 compares the adsorption performance of the geopolymer adsorbents in this study with other adsorbents. It was observed that the binding performance of the eco-adsorbents in this study for CV was ultrahigh compared with that reported by other adsorbents. This difference is linked to the synergistic effect of the adsorbents and the high active functional group density.

**Table 7:** Comparison between adsorption values for different CV by using different adsorbents.

Adsorbent	Maximum capacity (mg/g)	Reference
Charred rice husks (CRH) Xanthated rice husks (XRH)	62.85 90.02	(Homagai et al. 2022)^74^
Fly ash-based porous geopolymer	45.454	(Purbasari et al.2023)^35^
Zr-Ce-SBA-15	105	(Xiang et al., 2022)^75^
Nano porous carbon (NC)	3.3 to 19.1	(Nenyoo et al., 2023)^76^
InVO4	134.25	(Huang et al., 2023)^77^
Ch-IL@SPEEK composite	77.66	(Yılmazoğlu et al., 2024)^78^
SM1	227.27	Present study
SM2	277.78	Present study
SM3	322.58	Present study

##### Limitation of these study

One main limitation of our study is the examination of Moringa Seed Powder-modified slag-based geopolymer composites for their effectiveness in the regeneration and reuse as adsorbents for Crystal Violet (CV) dye. The recovery and regeneration of geopolymer materials after dye uptake requires additional treatment methods^73,79^. During the dye adsorption process, physical and chemical changes can occur, which may compromise their effectiveness^73,79^. Therefore, efficient regeneration strategies are necessary. However, these strategies can be complex and resource-intensive, highlighting the need for further research to ensure sustained performance without significantly degrading the adsorption capacity.

In our future research, we aim to enhance the specific surface area of geopolymer composites made from agricultural and industrial wastes. Additionally, we will develop robust regeneration techniques to promote environmentally sustainable dye adsorption and remediation.

## Conclusions

In the current study, a modified geopolymer was prepared with a combination of industrial and agricultural wastes (slag, and moringa seed powder). Furthermore, we examined the environmental application of a geopolymer binder as a crystal violet adsorbing agent for wastewater cleaning. The maximum compressive strength was 73.55 Mpa, obtained by using composite specimens weighing 0.2 weight% MSP, whereas mixes with a greater MSP content, 0.6 and 1 wt% gave lower compressive strength values. This result was incompatible with XRD and FTIR analysis. The performance of geopolymer on the elimination of CV dye from water was optimized using the batch adsorption method. Nitrogen adsorption/desorption isotherms give type IV isotherms with H3 hysteresis loops. The presence of moringa seed powder effectively enhances the surface area and pore volume of modified geopolymer. The adsorption technique’s results for the created geopolymer mixtures revealed that SM3 (1 wt% MSP) had enhanced the surface area and gave higher adsorption activity than other mixes toward crystal violet. Optimization conditions for the maximum removal efficiency of CV and adsorption capacity were obtained by pH 8, contact time 30 min, adsorbent dosage 0.01 g L^− 1^, and initial concentration of dye 10 mg L^− 1^. According to the isotherm and kinetic data, the Freundlich model and pseudo-second-order were supported by this adsorption interaction process. The XRD, FTIR, pore size analysis and microstructure findings also demonstrated the existence of crystal violet adsorbed on the surface of the geopolymers. These results have significant implications for the reuse of both agricultural and industrial waste, the removal of harmful substances from wastewater, and the enhancement of environmental sustainability. Based on these findings, future research can explore the potential and applicability of these geopolymer samples in large-scale applications. Furthermore, future research might focus on refining and regeneration of these types of geopolymers (containing agricultural waste) synthesis methods to increase their adsorption capability and reusability for various pollutants. Moreover, examining the long-term stability and effectiveness of these eco-friendly geopolymer samples in real-world wastewater treatment scenarios would be advantageous for practical applications.

**Fig. 15:**
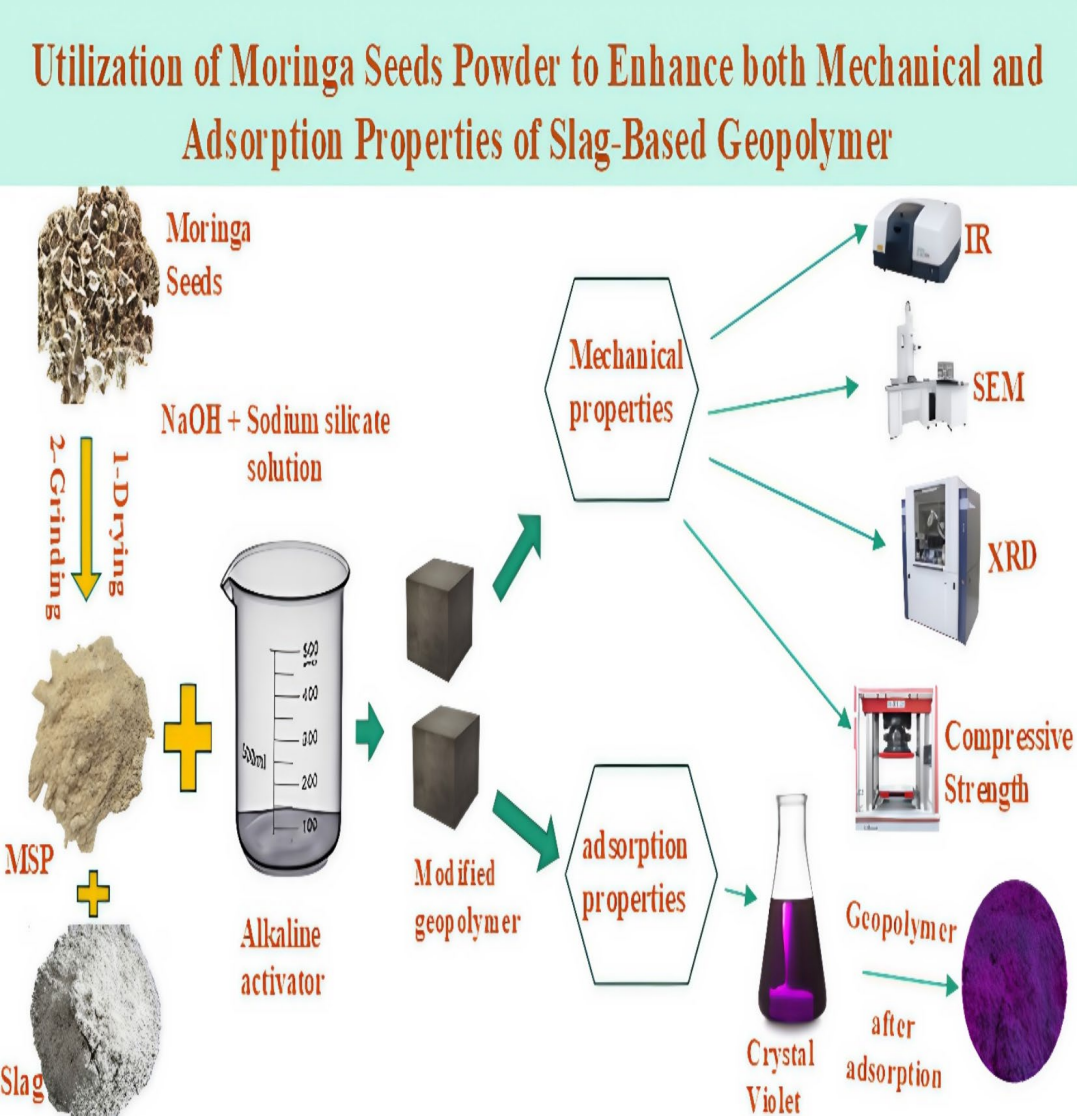
The schematic diagram of our study.

The summary of our study can be described in schematic diagram shown in Fig. 15.

## Data Availability

The authors declare that the data supporting the findings of this study are available within the paper and its Supplementary Information files. Should any raw data files be needed in another format they are available from the corresponding author upon reasonable request. Source data are provided with this paper.
